# Endometrial receptivity tests in reproduction: a SWOT analysis

**DOI:** 10.1016/j.xagr.2023.100260

**Published:** 2023-08-11

**Authors:** Juan A. Garcia-Velasco, Joaquín Llácer, Antonio Requena, Miguel Ángel Checa, José Bellver, Ernesto Bosch, Juan José Espinós, Francisco Fabregues, Ana Isabel Ortega, Juan Fontes, Spanish Infertility SWOT Group

**Affiliations:** 1 IVIRMA Global Research Alliance, IVIRMA Madrid, Madrid, Spain (Dr Garcia-Velasco); 2 IVIRMA Global Research Alliance, Ginefiv, Madrid, Spain (Drs Llácer and Requena); 3 Hospital del Mar-Parc de Salut Mar, Barcelona, Spain (Dr Checa); 4 Fundación Fertty, Barcelona, Spain (Drs Checa and Espinós); 5 Facultad de Medicina, Departamento de Pediatría, Obstetricia y Ginecología, Universidad de Valencia, Valencia, Spain (Dr Bellver); 6 IVIRMA Global Research Alliance, IVIRMA Valencia, Valencia, Spain (Drs Bellver and Bosch); 7 IVIRMA Global Research Alliance, IVI Foundation, Instituto de Investigación Sanitaria La Fe, Valencia, Spain (Dr Bellver); 8 Universidad Autónoma de Barcelona, Bellaterra, Spain (Dr Espinós); 9 Institut Clinic Gynecology, Obstetrics and Neonatology, Hospital Clinic, Barcelona, Spain (Dr Fabregues); 10 Grupo Mayo, Barcelona, Spain (Dr Ortega); 11 Hospital Universitario Virgen de las Nieves, Granada, Spain (Dr Fontes); 12 Instituto AVANTIA de Fertilidad, Granada, Spain (Dr Fontes); 13 Instituto de Investigación Biosanitaria IBS Granada, Granada, Spain (Dr Fontes)

**Keywords:** Endometrial receptivity test, infertility, window of implantation, SWOT

## Abstract

Endometrial receptivity and its management in assisted reproduction is now a significant focus of research interest. Endometrial receptivity tests, which analyze different panels of gene expression, are usually offered in fertility clinics to determine the women's individual ‘window of implantation’, providing a personalized timing for embryo transfer. However, there are still no definite indications on whether its inclusion in the study of the infertile couple or the study of patients with repeated implantation failure is essential.

## Introduction

Endometrial receptivity (ER) and its management in assisted reproduction technology (ART) is now a significant focus of research interest. ER tests (ERts), which analyze different panels of gene expression, are usually offered in fertility clinics to determine the women's individual “window of implantation” (WOI), providing a personalized timing for embryo transfer. However, there is still no definite indication of whether its inclusion in the study of the infertile couple or the study of patients with repeated implantation failure (IF) is essential.

## Methods

In this discussion article, based on a strengths, weaknesses, opportunities, and threats analysis, the different aspects of the application of ERt (ERA, Win-Test, rsERT test, ERPeak, ERMap, beREADY, and Tb-ERA test) in reproduction are evaluated following Oxford criteria (Oxford Centre for Evidence-Based Medicine).

## Strengths

ERts were developed as an attempt to identify the optimal ER status (WOI) in a more precise way than traditional endometrial dating, not only reaching this objective (evidence 2b)^1^ but also proposing a temporally optimal transfer time or personalized ET (pET) for each woman (evidence 2b)^2^ (Figure).

**Figure:**
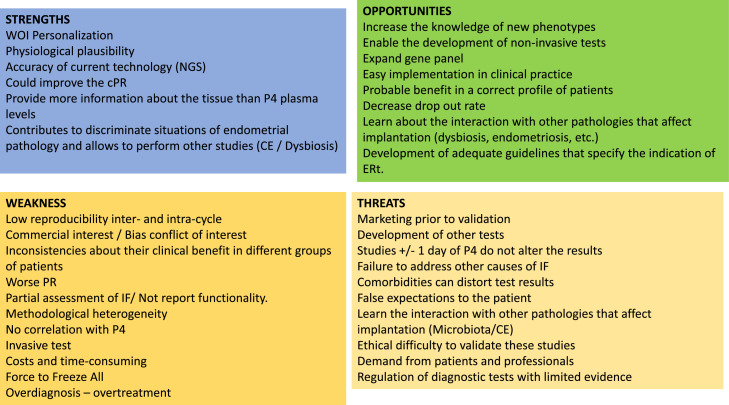
A SWOT analysis *CE*, chronic endometritis; *cPR*, cumulative pregnancy rate; *ERt*, endometrial receptivity tests; *IF*, implantation failure; *NGS*, next-generation sequencing; *P4*, progesterone; *PR*, pregnancy rate; *SWOT*, strengths, weaknesses, opportunities, threats; *WOI*, window of implantation.

García-Velasco et al. Endometrial receptivity tests in reproduction. Am J Obstet Gynecol Glob Rep 2023.

The first study on ER was published 10 years ago using the first developed ERt, the ERA (evidence 2b).^3^ The objective was to generate a genomic diagnostic tool to define the transcriptomic signature of an optimal human ER status in women with a long history of failed in vitro fertilization (IVF) cycles and repetitive IF (RIF) (evidence 2b).^4^ Henceforth, the analysis of ER-specific genes has increased exponentially, allowing to determine the relationships between the differentially expressed genes in the endometrium during the WOI and reproductive hormone level disturbances (evidence 2c)^5^ providing an endometrial assessment tool for accurate endometrial progression dating (evidence 2c)^6^ and identifying possible endometrial function dysregulations (evidence 2a)^7^ and phenotypes (evidence 2c).^8^

ERts have been suggested to improve cumulative pregnancy rate (cPR) as it was shown in a multicenter, prospective, randomized controlled trial (RCT) that included 458 patients undergoing their first and second IVF cycles, which compared the efficacy of performing the embryo transfer guided by either the results of the ERA test or according to the standard clinical care. No benefit was observed in the intention-to-treat analysis; however, when results were analyzed per protocol, the pregnancy rate (PR) and cPR at 12 months improved when guided by ERA (evidence 1b).^9^ Surprisingly, there was a 50% dropout in the study, and some methodological concerns have been mentioned.

Test-based technology is rapidly evolving. Microarray and polymerase chain reaction-based clinical tests are being replaced by next-generation sequencing, a more precise technique in quantifying global gene expression profiles and more comprehensive in coverage.

Moreover, a remarkable strength in the application of these tests is that endometrial transcriptomics has proven more objective and concordant with ER than serum progesterone (P4) (evidence 2c),^10^ and their use serves to discriminate other pathologies of endometrial origin, such as endometriosis-associated infertility (evidence 2a),^7^ and to perform other studies (chronic endometritis [CE] or dysbiosis).

## Weakness

One of the main limitations of these tests might be the need for more solid evidence to support the intra- and intercycle reproducibility within the same patient. The human endometrium is a dynamic tissue that experiences molecular and morphologic modifications throughout the menstrual cycle. Because of this extensive intercycle variation in reproductive physiology, the receptive status of the endometrium when the test is performed might differ from that of the embryo transfer cycle. ERA is one of the few ERts that has demonstrated reproducibility in published data on 7 patients (evidence 4),^1^ and this limited sample size may not represent the entire infertile population.

However, regarding other ERts, there are data on the reproducibility of ERMap from a double analysis of biopsies in 29 patients showing good reproducibility (evidence 3b).^11^

The methodological heterogeneity (dataset characteristics, algorithms, the technology employed for measuring gene expression, etc.) and the lack of agreement on the genes to be studied limit the standardization of ERt (evidence 2b).^6^ The overlap among the studies is relatively small. However, the signatures (different combinations of genes) disagreed in gene content in terms of gene intersections and functional concordance (evidence 3b).^8^

Another important limitation is that most ERt studies—at least those showing a positive result—are sponsored by the companies selling the test.^12^ Recently, independent studies that contradict previous sponsored studies and do not support the routine use of ERt to guide embryo transfer are being published.^13^

In addition, bearing in mind the published literature, there is still no demonstrated evidence regarding the usefulness of these tests in any specific group of patients with infertility. For example, in patients with RIF, pET using ERA does not seem to improve gestational outcomes compared with standard ET (evidence 2a).^13^ Furthermore, a displaced “window of receptivity” is shown to be the only endometrial dysfunction underlying RIF (evidence 2b),^14^ and ERA did not seem to distinguish between those with and without a history of IF (evidence 3b).^15^ More recently, a RCT not included in a previous meta-analysis conducted in patients with a good prognosis for IVF yielded a euploid blastocyst; moreover, even a potential negative effect of receptivity timing for transfer was suggested compared with standard timing (evidence 1b).^16^ Similarly, in patients with a donor cycle, pET guided by ERA was not recommended as poorer implantation rate (IR) and clinical PR have been reported (pooled data of the IR [relative risk (RR), 0.57; 95% confidence interval (CI), 0.36–0.92] and clinical PR [RR, 0.59; 95% CI, 0.41–0.85]) (evidence 2a).^12^^,^^17^

Of note, endometrial sampling for the ERt is considered to be an invasive procedure.^11^ Currently, ERA-guided pET increases the cost, forces to freeze all treatments, and delays IVF treatment (evidence 3b).^17^

Another limitation of the published studies is not considering embryo quality. Among all the available studies, none describes embryo policy regarding transfer, and there might be a possibility that receptivity tests perform differently in good vs poor embryo quality.

Finally, the risk of overdiagnosis and overtreatment is also present as the transcriptomic signature of the endometrium may exhibit more monthly variability than previously reported, leading to inaccurate and harmful recommendations for progesterone exposure.

## Opportunities

Transcriptomics offers the opportunity to obtain further molecular information that permits distinguishing new patterns of endometrial pathology or processes, thereby providing new endometrial taxonomies (evidence 2b)^18^ as molecular disruptions and molecular displacements. Both pathology (disruption) and asynchrony (displacement) have been identified in IF (evidence 3b).^8^ Moreover, the new generation of transcriptomic endometrial dating can offer accuracy for staging the endometrial tissue (evidence 2b),^19^ is easily implemented in clinical practice, can serve to reduce the number of dropouts, and could be the cornerstone for the development of noninvasive tests.

The expansion of genes implicated in different biological terms (endometrial cell division and proliferation, cell signaling and response, extracellular organization and communication, maternal immune response, etc.) could favor its implementation and its use in a correct patient profile (eg, patients with truly narrow WOI) where these tests might provide information that could be crucial.

A Grading of Recommendations Assessment, Development, and Evaluations–based assessment recommended a careful study of ER in ART cycles, distinguishing between couples undergoing ART for the first time and couples suspected to have RIF, even in the presence of a morphologically normal uterus. Furthermore, developing appropriate guidelines specifying the indication of ERt is a priority, and the treatment offered should be evidence based and designed to improve ER.

## Threats

Personalized treatment for endometrial factors has taken off, altering the clinical practice of more than 4000 reproductive clinics in more than 90 countries worldwide (evidence 5).^10^ However, this high degree of use mandates the clinician to consider the benefit and harm this test can inflict on the clinical outcomes of patients.

ERts are very limited in addressing other causes of IF and are considered a laboratory-developed test intended for use only in the laboratories in which they were created. In addition, most of the signatures were published for molecular investigation, but they have been translated to clinical practice with no previous validation (evidence 2b).^3^ Therefore, other tests, such as uterine peristalsis or the status of nucleolar systems, are being proposed as alternatives to ERt to determine the chance of clinical pregnancy before embryo transfer.

P4 exposure has been assumed to be able to better or postpone the endometrial maturity by precisely 12-hour intervals.^11^ However, this concept is not backed by studies showing that plus or minus 1 day in P4 exposure does not affect implantation (evidence 1b),^20^ questioning the established importance of ±12 to ±24 hours.

In addition, comorbidities may distort test results (eg, endometriosis) as they may have negative effects on the individual WOI, leading to embryo-endometrial asynchrony; therefore, the diagnosis and treatment of these conditions, such as CE, should be performed before ERA testing (evidence 2b).

Finally, although there is limited supporting evidence for this add-on treatment in IVF, some patients with infertility assume that they are not receiving the most advanced care if a test is not offered or performed. Therefore, there is substantial demand from patients, physicians, and the biotechnology industry (evidence 3b) with limited information regarding their regulation for clinical use and a significant ethical difficulty in validating these studies.

## Conclusion

Prevailing evidence does not support the use of ERt daily. Additional well-designed research is required to ascertain the efficacy of ERt before achieving wider usage.
